# Changes in the Bio-Compounds and Biological Activities of Eight Whole Grains Fermentation Starter with Different Oxidized Chin-Shin Oolong Teas

**DOI:** 10.3390/foods12081643

**Published:** 2023-04-14

**Authors:** Chih-Feng Wang, Cui-Rou Huang, Ying-Chen Lu

**Affiliations:** Department of Food Science, National Chiayi University, #300 Xuefu, Chiayi City 600, Taiwan

**Keywords:** *Acetobacter pasteurianus* LYC1783, angiotensin converting enzyme inhibition, antioxidants, Chin-shin oolong, GABA, two-step fermentation

## Abstract

Chin-shin oolong tea is the most widely planted variety in Taiwan. This study fermented eight whole grains fermentation starter (EGS) with light (LOT), medium (MOT), and fully (FOT) oxidized Chin-shin oolong teas for ten weeks. Comparing the three fermentation beverages, it was found that LOT fermentation can obtain the highest catechins (1644.56 ± 60.15 ppm) among the functional and antioxidant components. MOT can obtain the highest glucuronic acid (19,040.29 ± 2903.91 ppm), tannins, total phenols, flavonoids, and angiotensin-converting enzyme (ACE) inhibitory activity. FOT can obtain the highest GABA (1360.92 ± 123.24 ppm). In addition, both the LOT and MOT showed a significant increase in their ability to scavenge DPPH radicals after fermentation. EGS fermented with lightly or moderately oxidized Chin-shin oolong tea may be considered a novel Kombucha.

## 1. Introduction

Tea from *Camellia sinensis* is the second most popular beverage in the world, after water [1]. Tea can mainly be divided into three categories, according to its oxidation degree (the release of polyphenol oxidase and peroxidase): unoxidized (green tea), partially oxidized (oolong tea), and fully oxidized (black tea). Tea leaf oxidation and fermentation is usually followed by a drying process to stop the enzymatic reactions in the fermentation [2]. Research conducted in clinical settings has revealed that tea possesses both preventive and therapeutic properties for oxidative stress-related illnesses, such as cardiovascular disease, liver disease, type 2 diabetes, cognitive dysfunction, and cancer [3].

Chin-shin oolong tea, a popular tea cultivar, is extensively grown in Taiwan at elevations above 1000 m. While Chin-shin oolong tea can be processed into different levels of oxidation, it is typically lightly oxidized to preserve its delicate floral aroma and sweet taste. However, some producers may choose to process it into moderately or fully oxidized tea to create a different flavor profile. Regardless of the level of oxidation, Chin-shin oolong is highly valued for its fragrant taste and is considered one of the top oolongs produced in Taiwan.

Kombucha is a traditional beverage produced by a consortium of symbiotic microorganisms, primarily composed of acetic acid bacteria (AAB) and osmophilic yeast [4]. While it has recently gained more global popularity, Kombucha was first consumed in China more than 2000 years ago. Kombucha is known for its various functional properties, which include antioxidant activity, the inhibition of angiotensin-converting enzyme (ACE), liver detoxification, and antimicrobial, anticancer, and antimutagenic effects. These properties are thought to be due to the presence of polyphenols, glucuronic acid, lactic acid, acetic acid, amino acids, antibiotics, tannins, and micronutrients that are produced during the fermentation process [5,6,7].

Kombucha also contains γ-aminobutyric acid (GABA). GABA is an important neurotransmitter; it inhibits nerve transmission, calms a stressed-sympathetically active nervous system, and has anti-hypertensive effects [8]. Tea rich in GABA has been shown to lower blood pressure [9]. It down-regulates the immune response, reducing the release of inflammatory compounds [10]. The results from a short-term intervention study indicate that the regular consumption of green, black, or oolong tea may lower the risk of developing high blood pressure [11].

The traditional method of preparing Kombucha involves adding a symbiotic colony of bacteria and yeast (SCOBY) to tea brewed with (mainly white) sugar. Allowing the mixture to ferment elicits the drink’s desired ingredients and nutritional properties [12]. However, sugar is low in nutrition, and excessive sugar intake can quickly induce obesity. The use of eight whole grains as a starter for two-step fermentation can effectively enhance the functional bioactive compounds and antioxidants [13]. As such, this study used an eight whole grains fermentation starter (EGS), instead of sugar, as a fermentation starter. EGS provides carbohydrates for the bacteria responsible for the fermentation process and the acidity needed to inhibit the growth of harmful bacteria. Furthermore, in contrast to Kombucha, which is mainly fermented with a black tea decoction, this study fermented tea leaves using Chin-shin oolong tea. At present, there is limited research available on the biological activities and composition of Chih-shin oolong tea. Additionally, no studies have been conducted on tea fermentation using Chin-shin oolong tea leaves as the raw material with EGS. This study examines the changes in the biological components and biological activities of EGS fermented with different oxidized Chin-shin oolong teas (lightly, moderately, and fully oxidized tea) for ten weeks.

## 2. Materials and Methods

### 2.1. Chemicals and Reagents

The following chemicals were obtained from the specified suppliers: L-ascorbic acid from Riedel-de Haën (Seelze, Germany), caffeine, vanillin, and NaCl (Sodium chloride), from Sigma Aldrich (Saint Louis, MO, USA), quercetin from the Tokyo Chemical Industry Co. (Osaka, Japan), methanol from Macron Fine Chemicals (Center Valley, PA, USA), NaOH (Sodium hydroxide), acetonitrile, and gallic acid from Alfa Aesar (Santa Ana, CA, USA), and o-phosphoric acid were purchased from Honeywell (Muskegon, MI, USA), and (+)-catechin from Toronto Research Chemicals (Toronto, ON, Canada). The reagents utilized in the investigation were of the utmost purity commercially available.

### 2.2. Sample Preparation and Fermentation Method

The Chin-shin oolong tea variety, produced in Alishan, Taiwan, was used for the fermentation. The tea samples were purchased from the Bai Feng Co. in Chiayi, Taiwan. To prepare the EGS, 5000 g of grains (each weighing 625 g) were mixed with 20 L of deionized water in a jar. The jars were agitated twice daily and kept at 30 °C for a duration of four months. Following filtration, the mixtures were stored at room temperature for subsequent analysis.

Five hundred grams of each tea leaf variety (lightly oxidized, moderately oxidized, and fully oxidized) was mixed with a 6600 mL fermentation starter and 500 mL RO water. Each tea leaf fermentation was duplicated so that there were two samples of each tea variety. Six identical 10 L glass jars were used for a 10 week fermentation process, carried out at a temperature of 30 °C. Daily stirring was performed on each jar, and triplicate solution samples of 50 mL were collected every week. These samples were filtered, centrifuged, and subsequently stored at a temperature of −20 °C for further experimentation.

### 2.3. Culture and Identification of Acetic Acid Bacteria

A sample of 0.1 mL was mixed in 0.9 mL Hestrin-Schramm (HS) broth and diluted at the appropriate dilution rates. Sequential decimal dilutions of the samples from the different tea beverages were plated in duplicates on HS agar and incubated at 25 °C for 48 h.

The PCR procedure involved the use of 16S primers (16F: GTATTACCGCTGCTG/16R: AGAGTTT-GATCCTGGCTCAG). The amplification of DNA fragments was achieved through an initial heating step at 95 °C for 5 min, followed by 35 amplification cycles. Each cycle consisted of a denaturation step at 94 °C for 1 min, an annealing step at 36 °C for 1 min, and an extension step at 72 °C for 1 min. Sequencing of the PCR products was carried out at The National Cheng Kung University Center for Genomics Medicine.

### 2.4. Total Phenolic Content (TPC) Assay

The method for determining the total phenolic content concentration in the crude extract was modified from a previous study [14]. The addition of 25 μL of the test solutions to 1.0 mL of 2% Na_2_CO_3_ was performed, followed by the addition of 0.25 mL of Folin-Ciocalteau reagent (50% concentration). The mixture was incubated at 25 °C for 30 min before measuring the 750 nm absorbance value using a spectrophotometer (Meter-tech SP-830 Plus, Taipei, Taiwan). To prepare the blank sample, all of the reagents and solvents were combined, excluding the standards or the test compounds. Gallic acid was used as the standard and prepared in concentrations ranging between 0.001 mg/mL and 1.0 mg/mL. Using a gallic acid standard curve, the TPC value was finally determined and expressed in µg GAE/g.

### 2.5. Total Flavonoid Content (TFC) Assay

By using a modified approach, the TFC of the extracts was determined [15]. Methanol (0.8 mL), 10% aluminum chloride (400 µL), 1 M potassium acetate (400 µL), and redistilled water (200 µL) were mixed with a 0.2 mL sample of the properly diluted extract. The reaction mixture was incubated for 40 min at 25 °C, and its absorbance was measured at 415 nm using a spectrophotometer (Meter-tech SP-830 Plus, Taipei, Taiwan).

### 2.6. ABTS Radical Scavenging Assay

The ABTS assay for measuring the radical scavenging activity was performed using a method that was modified based on the procedure described in [15]. The ABTS solution was obtained by mixing 4.9 mM K_2_S_2_O_8_ with 20 mL of deionized water and 14 mM of ABTS salt, which was stored in a dark bottle and refrigerated. After diluting the stock solution with deionized water, the ABTS+ solution was left to incubate in the dark at room temperature for 16 h. The radical solution was analyzed with a spectrophotometer (Meter-tech SP-830 Plus, Taipei, Taiwan) until the baseline absorbance reading of 0.7 ± 0.005 at 734 nm was attained.

To measure the radical scavenging ability, 0.05 mL of the sample was mixed with 1.95 mL of the diluted ABTS solution, and the mixture was incubated for 6 min. The spectrophotometer was used to measure the absorbance at 734 nm using deionized water as a blank. The decrease in absorbance at 734 nm was calculated to determine the level of inhibition, and the results were plotted as a function of the concentrations of antioxidants and Trolox, which were used as a standard curve.

### 2.7. DPPH Radical Scavenging Assay

The ability of the tested extracts to neutralize the steady DPPH radical was evaluated by determining the absorbance with a spectrophotometer (Metertech SP-830 Plus, Taipei, Taiwan). To perform the DPPH radical scavenging assay, a DPPH solution methanol (with an absorbance of approximately 1.2) was mixed with 0.4 mL of the sample in a 1 mL cuvette. The samples were kept in the dark at room temperature for 50 min, followed by measuring the absorbance at 517 nm with deionized water for reference [15]. Ascorbic acid was used as the standard.

### 2.8. pH and Total Titratable Acidity (TTA) Determination

The pH meter was utilized to measure the pH value of each sample (pH 2–11, Hanna, Padova, Italy). Simultaneously, TTA analysis was conducted using the procedure described by [16]. In brief, 1 mL of an aqueous extract of each sample was dissolved in 20 mL of distilled water, and a 0.1 M NaOH solution was used to titrate the resultant solution. During the titration process, 1% phenolphthalein was utilized as an indicator, and the solution was continuously stirred until the first visible color change to pink was observed. The results matched the expected outcome in terms of the concentration of acetic acid present in the sample.

### 2.9. Analysis of Organic Acid, Carbohydrates, Caffeine, Catechin, and Ethanol

The filtered samples were analyzed using high performance liquid chromatography (HPLC) systems, utilizing an injection volume of 10 μL for the filtrate. The content of lactic acid, acetic acid, and glucuronic acid was quantified by means of a UV detector (210 nm) and a C-18 column (5 μm, 4.6 mm × 250 mm; Hitachi High-Tech Fielding Corporation, Tokyo, Japan). The analysis was carried out at a flow rate of 0.6 mL/min, with a duration of 40 min at 30 °C. The mobile phase was comprised of a blend of 0.05 M phosphate buffer, and acetonitrile in a ratio of 90:10 *v*/*v*. Prior to the analysis, filtration of the samples was carried out using a sterile microfilter with a pore diameter of 0.22 μm to ensure sterility.

To determine the content of glucose, fructose, and ethanol, an Agilent Hi-Plex H ion exchange column (300 mm × 7.7 mm) with an RI detector was used at a flow rate of 0.6 mL/min and a running time of 40 min at 30 °C. The mobile phase comprised 100% 0.05 M H_2_SO_4_.

The catechin content was analyzed using a C-18 column with a UV detector. The HPLC conditions included mobile phase A: acetonitrile and phase B: 3% acetic acid. The gradient program applied was described in the following manner: from 0 to 27 min, the eluent composition was 37%; from 27 to 29 min, it was 95% B. The eluent flow rate was 0.5 mL/min, and the column temperature was maintained at 35 °C. During each analysis, 10 μL of the sample was injected and UV detection was carried out at a wavelength of 280 nm [17].

The analysis of the caffeine content was conducted using a C-18 column employing a UV detector. The HPLC conditions included mobile phase A: acetonitrile and phase B: deionized water. The gradient program used in the experiment was as follows: for the first 0–10 min, the eluent consisted of 85% of solvent A, and for the next 10–20 min, the eluent was composed of 20% of solvent B. The column was kept at 35 °C, and the eluent was flown through at a rate of 1 mL/min. The sample was injected in a volume of 10 μL, and UV detection was performed at a wavelength of 280 nm.

### 2.10. GABA Measurements

To prepare the stock standard solutions of GABA (1000 ppm), the standards were dissolved in individual 10 mL volumetric flasks, which were then kept in the dark. For each measurement, working solutions of 62.5, 125, 250, 500, and 1000 ppm were created by diluting the stock solutions with deionized water. Each prepared sample (1 mL) was supplemented with 2-hydroxynaphthaldehyde (0.3% *w*/*v*) in methanol, then 0.5 mL of boric acid-NaOH buffer (pH 8.5) was added to a 5 mL volumetric flask. The resulting mixture was subjected to heating at 85 °C for 15 min using a temperature-controlled heating device, followed by cooling it to room temperature. Adjusting the solution to 5 mL with methanol, it was then kept at 4 °C until analysis, in accordance with the instructions provided in [18,19].

To analyze the GABA content, the samples were passed through a sterile microfilter with a pore size of 0.22 μm. Next, 10 μL of the filtered sample was injected into an HPLC system (Shimadzu sci-10a, Kyoto, Japan).

### 2.11. ACE Inhibition Activities Assay

A previous method was modified to determine the ACE inhibition activity of the samples [20]. In each test, a total volume of 60 μL was prepared by combining 15 μL of Hippuryl-Histidyl-Leucine (0.1 M potassium phosphate buffer containing 4 mmol/L of Hip-His-Leu), 30 μL of ACE, 6 μL of the sample, and 9 μL of 0.1 M potassium phosphate buffer (buffered with 0.3 M NaCl at pH 8.2). The solution was then incubated at 37 °C in a deionized water bath for 1 h. To stop the reaction, 50 μL of 1 N HCl was added. The mixture was cooled in an ice bath and then treated with 50 μL of BSC and 100 μL of pyridine as coloring agents. After shaking, the mixture was rapidly returned to room temperature. After transferring 200 μL of the solution to a 96-well plate, the absorbance was measured at 410 nm using an absorbance spectrophotometer (BMG LABTECH, SPECTRO Star Nano, Offenburg, Germany). The IC_50_ value, which represents the concentration of inhibitor needed to inhibit 50% of the ACE activity, was calculated.

### 2.12. Condensed Tannins Assay

According to the previous method [21], 125 μL of the sample, 375 μL of HCl, and 750 μL of 4% vanillin were combined and allowed to react in the dark for 20 min. A spectrophotometer measured the sample’s absorbance at a wavelength of 500 nm. A calibration curve was prepared using catechin standards at concentrations ranging between 0.001 mg/mL and 1.0 mg/mL, and the condensed tannin content of the samples was determined by comparing their absorbance with the calibration curve.

### 2.13. Statistical Analysis

All of the analyses were conducted in triplicates. The results are expressed as mean ± standard deviation (SD). A one-way ANOVA was employed to analyze the statistical significance of the data. To compare the groups, Duncan’s multiple-range tests were conducted. The statistical analysis was performed using SPSS Statistics v.10 software (IBM Corp., New York, NY, USA), and a significance level of *p* < 0.05 was selected. Sigma Plot 10 was used to perform all of the graphical representations.

## 3. Results and Discussion

### 3.1. Variable Bacteria Counts and Identification after Fermentation

**Figure 1:**
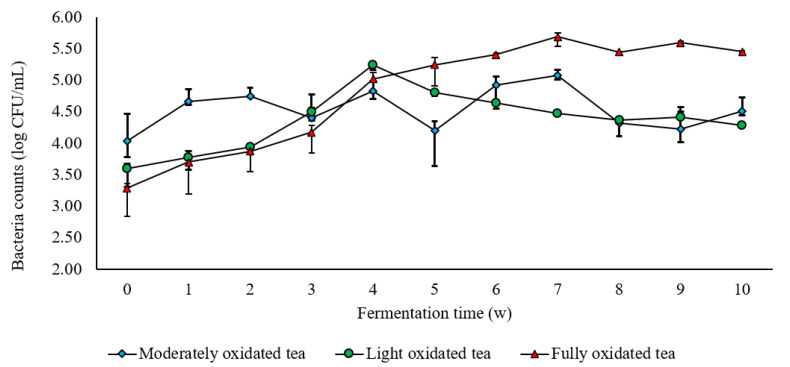
The variation in bacteria counts of *Acetobacter pasteurianus* during the fermentation of different oxidized teas. The mean values ± standard variation of three replications indicated a significant difference at *p* < 0.05.

During the fermentation process, the acetic acid bacteria of the three teas showed an upward trend (Figure 1). One acetic acid bacterium was isolated and identified as *Acetobacter pasteurianus* LYC1783 in the final fermentation products. *Acetobacter pasteurianus* plays an important role in acetic fermentation, and acetic acid has been shown to have good antimicrobial activity against microorganisms [22,23]. This indicates that yeast and lactic acid bacteria are not significantly involved in fermentation. Furthermore, the phenols and tannins extracted from the tea exhibited a wide-ranging ability to inhibit both Gram-negative and -positive bacteria [24]. In addition, the pH of the grain fermentation starter was low and the acidity was high (pH 3.4, acidity 4.97). A low pH inhibits the growth of yeast; hence, the ethanol production was minimal (Figure 1).

The bacterial number of the acetic bacteria in the LOT was 3.94 log CFU/mL in Week 2; the growth rate was only 0.17 log CFU/mL per week, slower than that of MOT and FOT. *A. pasteurianus* LYC1783 grew rapidly after the second week, reaching the highest CFU at 5.24 log CFU/mL (0.55 log CFU/mL per week) during Week 4. It gradually decreased to 4.28 log CFU/mL by Week 10.

*A. pasteurianus* LYC1783 grew the fastest in the first two weeks, where the average growth rate was 0.047 log CFU/week. It reached 4.75 log CFU/mL in the second week and then decreased in Week 3. After the third week, it reached 4.83 and 4.92 log CFU/mL in Weeks 4 and 6, respectively. A maximum of 5.08 log CFU/mL was reached at Week 7. The concentration rapidly decreased to 32,500 CFU/mL by Week 10. The average growth rate in the last three weeks was only −0.19 log CFU/mL per week, which was likely related to the 37.25% reduction in glucose during that period.

The growth rate of the bacteria in the first seven weeks in the FOT was faster than those of the other two fermented teas, reaching 0.34 log CFU/mL per week. The bacterial count reached its highest point at 5.69 log CFU in Week 7. The bacterial count of the FOT in Week 10 was 5.45 log CFU, much higher than the 4.28 log CFU in the LOT and 4.51 in the MOT. The decline in *A. pasteurianus* LYC1783 might be due to the lack of nutrients and increased environmental acidity [25].

**Figure 2:**
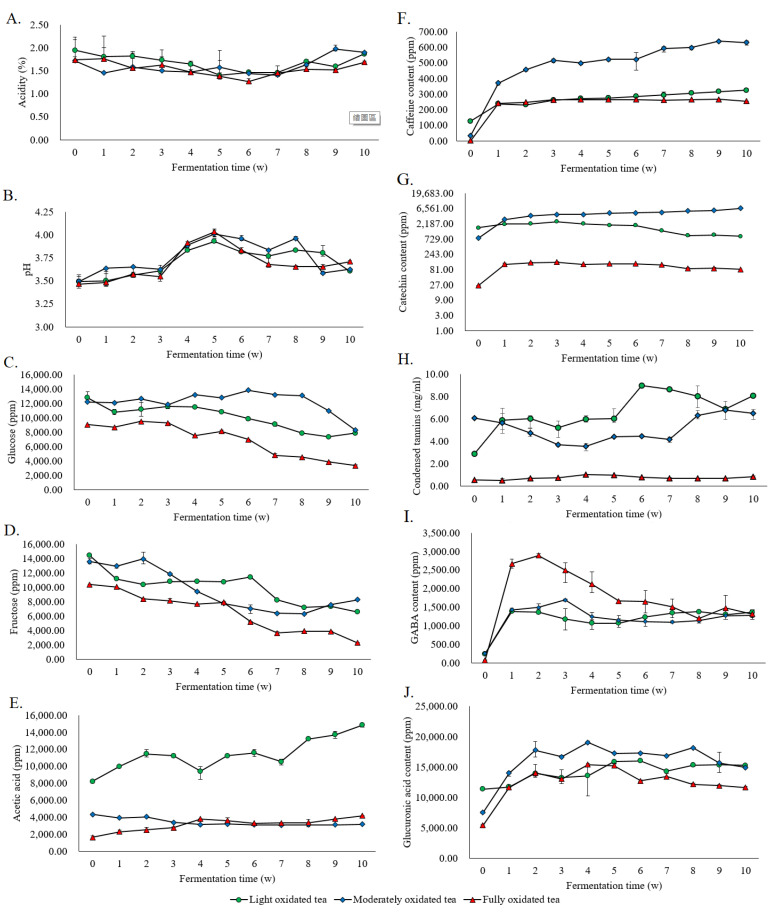
Changes in acidity (**A**), pH value (**B**), glucose (**C**), fructose (**D**), acetic acid (**E**), caffeine (**F**), catechin (**G**), condensed tannins (**H**), GABA (**I**) and glucuronic acid (**J**) during the fermentation process of different oxidized teas. The mean values ± standard variation of three replications indicated a significant difference at *p* < 0.05.

In the LOT and FOT fermentation samples, the growth rate of *A. pasteurianus* LYC1783 was slow in the first three weeks. During the fermentation process, the growth rate of the acetic bacteria in the FOT was the highest, followed by the MOT. The LOT was the least favorable environment for *A. pasteurianus* LYC1783. As such, the *A. pasteurianus* LYC1783 growth was optimal in the FOT during fermentation. The increase in caffeine might be related to the microorganisms. The slower increase in caffeine during the fermentation process of the FOT (Figure 2F) compared to the MOT and LOT could account for these results.

### 3.2. The Acidity, pH, Organic Acid, and Sugar

Figure 2A shows that the acidity of the LOT, MOT, and FOT decreased from the first week (1.45 ± 0.14%, 1.45 ± 0.14%, 1.76 ± 0.35%, respectively) to the seventh week (1.41 ± 0.02%, 1.41 ± 0.02%, 1.45 ± 0.11%, respectively). Then, it rose by the tenth week (1.90%, 1.90%, 1.69 ± 0.02%, respectively). The acidity of the three oxidized tea beverages increased in the late fermentation period. This is primarily due to the increase in acetic acid, glucuronic acid, catechin, caffeine, and other organic acids produced by acetic acid bacteria after metabolizing glucose (Figure 2). The pH values of the three oxidized tea beverages ranged between 3.47 and 4.04 during the ten weeks of fermentation (Figure 2B). The pH increases during fermentation were due to the alkaline components (caffeine, theophylline, amino acids) in the tea being released. Neither ethanol nor lactic acid was detected during the fermentation process in the LOT, MOT, and FOT samples.

The glucose in the LOT, MOT, and FOT was 12,778.81 ± 708.85 ppm, 12,182.13 ± 45.56 ppm, and 9031.20 ± 25.29 ppm, respectively, in the first week. It dropped to 7838.88 ± 23.04 ppm, 8275.68 ± 34.27 ppm, and 3350.31 ± 113.99 ppm, respectively, by Week 10. This is because the acetic acid bacteria metabolized the glucose and fructose into acetic acid during the fermentation (Figure 2C,D).

The LOT had the highest acetic acid among the three teas during fermentation, particularly from the seventh week to the end of the fermentation process. Hence, this process increased faster than in the other two teas (Figure 2E). The acetic acid of the MOT was similar to that of the FOT sample. However, the FOT was lower, at 1636.95 ± 166.23 ppm at the beginning, and slightly higher than the MOT in Week 4. The decrease in acetic acid was a result of acetic acid metabolism by acetic acid bacteria, converting it into acetyl-CoA [26].

The caffeine quantity increased in the LOT and MOT samples with an increase in the fermentation time. The caffeine content was the highest in the MOT, reaching 630.63 ± 14.92 ppm in the tenth week; this was much higher than the LOT, at 324.38 ± 7.34 ppm, and FOT at 255.23 ± 0.39 ppm (Figure 2F). The raw materials of the fermented tea were all fermented in jars. This may have caused the continual release of the tea’s caffeine by the acetic acid bacteria during fermentation. Microbial enzymes, produced by microorganisms, are secreted to the surface of tea leaves to catalyze the biosynthesis to produce caffeine. Alternately, caffeine is a secondary metabolite resulting from the microorganisms in tea leaves using components such as xanthine nucleosides [27]. The caffeine content in the FOT before fermentation was 1.59 ± 0.17 ppm less than the other two oxidized tea beverages (126.75 ± 1.19 ppm and 33.46 ± 0.19 ppm, respectively). There was little change in the caffeine concentration during fermentation. The fermentation of LOT and MOT produces more caffeine than that of FOT. Although clinical trials have shown that high caffeine consumption can promote weight loss through increased fat metabolism and thermogenesis [28,29], it is important to consider the effects of caffeine on the nervous system, which can be activating [30].

### 3.3. Variation of Functional Compounds

Before fermentation, the LOT had the highest catechin (1644.56 ± 60.15 ppm). It reached the maximum value of 2566.54 ± 28.19 ppm in the third week of fermentation and began to decrease slowly after the fourth week (Figure 2G). The decrease in catechin may be due to decomposition caused by microbial enzymes and autoxidation [31]. However, the MOT continued to increase to 1130.39 + 87.15 ppm by Week 10. The FOT had the least catechins, and the fermentation process increased from 25.56 ± 0.54 ppm to 81.66 ± 33.62 ppm, which may be due to an increase in theaflavin gallates and thearubigins after tea oxidation [32]. This indicates that, compared with the other two teas, LOT can obtain the highest catechins in the shortest time after fermentation. EGCG is the richest catechin, has the most phenolic hydroxyl groups, and has been reported to exhibit the most potent antioxidant activity among catechins, even stronger than vitamins C and E [33]. The tannin content before fermentation was the highest in the LOT (6.05 ± 0.14 ppm) and the lowest in the FOT (0.57 ± 0.03 ppm). The tannin value gradually decreased after the LOT fermentation and increased significantly in Week 8, reaching a maximum value of 6.79 ± 0.81 ppm in Week 9. The MOT was 2.91 ± 0.20 ppm before fermentation, much lower than the LOT. However, it continued to rise during fermentation, reaching a maximum value of 8.97 ± 0.12 ppm in the sixth week of fermentation, and then gradually decreased.

As a non-protein amino acid, GABA serves as the main inhibitory neurotransmitter in the mammalian brain for stress reduction and sleep [34]. The concentrations of GABA in the LOT and MOT samples before fermentation were similar (243.53 ± 10.35 ppm, 249.99 ± 5.39, respectively), whereas the FOT had the lowest (76.67 ± 3.60) (Figure 2I). The LOT fermentation process continued to increase and reached the highest value of 1360.92 ± 123.24 ppm in Week 10. This was higher than the MOT’s 1265.06 ± 61.38 ppm but similar to the FOT’s 1312.12 ± 100.95 ppm. The MOT reached the highest point, of 1692 ± 288.92 ppm, in the third week of fermentation and then decreased.

The glucuronic acid in the MOT and FOT increased rapidly in the first two weeks of fermentation. They peaked at 19,040.29 ± 2903.91 ppm and 15,384.64 ± 194.81 ppm, respectively, in Week 4, and then decreased to 14,930.53 ± 125.69 ppm and 11,734.43 ± 162.20 ppm, respectively. The LOT reached the highest concentration of 17,010.95 ± 92.32 ppm in Week 6 and slowly reduced to 15,259.804 ± 71.12 ppm by Week 10. The values in the LOT and FOT in the tenth week were very similar. However, FOT can reach the highest glucuronic acid in the shortest time. Moreover, the increase rate before and after fermentation in the FOT sample was 1532.50%.

### 3.4. Effect on the Antioxidant Capacity

**Figure 3:**
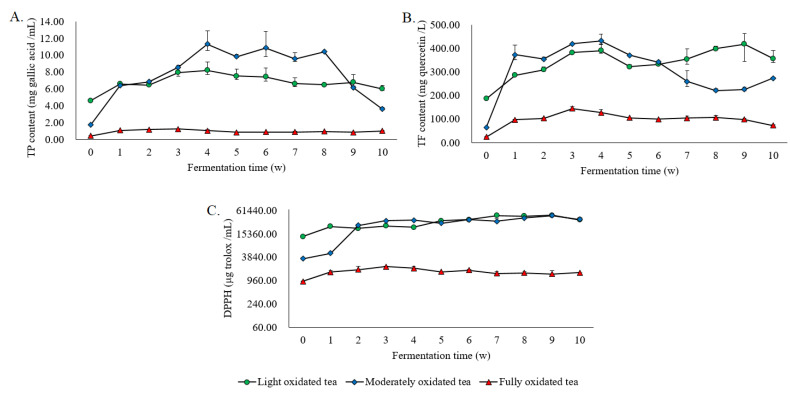
Changes in the antioxidant contents and capacity of three different oxidized teas during fermentation. (**A**) Total phenols (TP) content, (**B**) Total flavonoids (TF) content, (**C**) DPPH antioxidant capacity. The mean values ± standard variation of three replications indicated a significant difference at *p* < 0.05.

The LOT had the highest concentration of total polyphenols content (TPC) and total flavonoids content (TFC) before fermentation (4.62 ± 0.02 mg/mL and 186.83 ± 2.12 mg/mL, respectively). In contrast, the FOT sample had the lowest, at 0.43 ± 0.02 mg/mL and 24.13 ± 2.38 mg/mL, respectively. The TPC and TFC of the MOT were higher than those of the LOT (in Weeks 2 and 3), which were 6.84 ± 0.02 mg/mL and 372.80 ± 0.26 mg/mL, respectively (Figure 3A,B). The TPC and TFC reached their highest values during Week 4: 11.32 ± 0.70 mg/mL and 432.33 ± 17.46 mg/mL. Then, the TPC and TFC of the MOT dropped to 3.59 ± 0.29 mg/mL and 273.02 ± 23.81 mg/mL by Week 10, respectively.

The rise in flavonoids and catechins at the beginning of the fermentation in the three oxidized tea beverages is caused by TFCs (epicatechin isomers) being released from the acid-sensitive microbial cells, leading to higher concentrations [35]. The TPC and TFC production in the LOT sample reached the highest values of 8.19 ± 1.12 mg/mL and 417.78 ± 7.14 mg/mL at Weeks 4 and 9, respectively, then decreased to 6.01 ± 0.17 mg/mL and 356.83 ± 2.38 mg/mL, respectively. The values were still higher than the MOT (3.59 ± 0.29 mg/mL and 273.02 ± 23.81 mg/mL, respectively) and FOT (1.00 ± 0.04 mg/mL and 73.33 ± 2.38 mg/mL, respectively) at Week 10. The TPC and TFC of the FOT simultaneously reached their highest values of 1.22 ± 0.05 mg/mL and 145.26 ± 4.52 ppm in Week 3, but then gradually decreased. The TPC and TFC in the LOT decreased more slowly than in the other two oxidized tea beverages. This indicates that tea with a lower oxidation degree can maintain higher levels of flavonoids and total polyphenols. Among the three oxidized tea beverages, the LOT had the highest concentrations of TPC and TFC after ten weeks of fermentation. The TPC and TFC concentrations of these three oxidized tea beverages after fermentation were much higher than before fermentation. This indicates that the correlation between flavonoids and total polyphenols is positively associated with the degree of tea oxidation. Phenolic compounds are a diverse class of secondary metabolites found in natural products that are associated with a wide range of biological activities. These compounds have been shown to possess antioxidant properties that may help to reduce the risk of diseases related to oxidative stress. [36,37].

Regarding the DPPH’s performance ability to scavenge free radicals, the highest values obtained were 46,134.62 ± 480.77 μg/mL for the LOT at Week 9, 45,057.69 ± 19.23 μg/mL for the MOT at Week 9, and 2190.38 ± 21.15 μg/m for the FOT at Week 3 (Figure 3C). Although the DPPH scavenging ability of the LOT was more significant than that of the MOT and FOT before fermentation, the scavenging ability of the MOT’s DPPH after fermentation was nearly the same as the DPPH scavenging ability of the LOT (before fermentation). One possible reason the DPPH scavenging ability of FOT is lower is that its TFC and TPC are much lower than those of the LOT and MOT. The above results indicate that the total phenols and flavonoids of the LOT remained at a high level until the tenth week of fermentation. However, the performance of the MOT in DPPH scavenging free radicals was closer to the LOT between Weeks 2 and 10. The DPPH before and after MOT fermentation increased by 935.34%, much higher than the other two teas. Among the three oxidized tea beverages, the LOT and MOT obtained the highest antioxidant value in Week 9 of fermentation. In addition, the MOT obtained the highest TPC and TFC in Week 4. The performance of the FOT, whether in total phenols, flavonoids, or DPPH, was much lower than that of the LOT and MOT before and after fermentation. This may be because the antioxidant properties of black tea are reduced during the prolonged fermentation of polyphenol oxidase [38]. The antioxidant properties of the same variety of tea may decrease with an increased degree of fermentation [39]. The findings of this study are consistent with those reported in [12], which showed that green tea had the highest antioxidant potential (measured using DPPH), followed by white tea, with red tea exhibiting the lowest potential. The antioxidant activity of oolong tea was found to be intermediate between that of green and black teas, as measured using the same experimental methods [40]. Moreover, teas obtained from a single cultivar and grown under identical conditions exhibited decreasing antioxidant properties with the increasing degree of fermentation [39].

### 3.5. Effect on the ACE Inhibition Capacity

**Figure 4:**
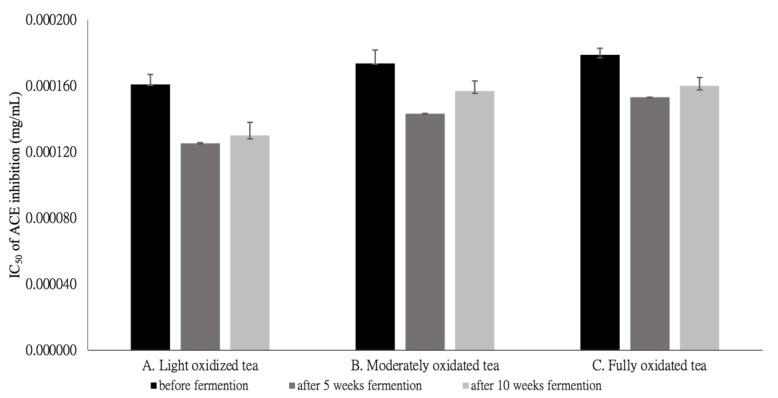
Effect of IC50 on ACE inhibition activity of Chin-shin oolong tea fermented with three different oxidized levels: before fermentation, after 5 weeks, and after 10 weeks.

Comparing the effects of IC_50_ on the ACE inhibition of the three teas during three fermentation periods, the performance after fermentation was better than before fermentation. The LOT performed the best, especially in Week 5. The IC_50_ inhibitory concentration at Week 5 of the LOT sample was 0.000125 mg/mL, lower than the 0.000161 mg/mL before fermentation, and slightly higher than the 0.00013 mg/mL at Week 10. The LOT performed best in Week 5, which was the case for the other two teas (Figure 4). The overall performance of the FOT’s ACE inhibitory ability is the worst before or after fermentation. The variation in the ACE inhibitory activity among teas processed by different methods can be attributed to the proportion of unoxidized catechins. Epigallocatechin 3-O-methyl gallate, which exhibits inhibitory effects on ACE, trypsin, and chymotrypsin, has been shown to be highly effective in reducing blood pressure [41]. The LOT had the highest content of catechins during fermentation, which may be the reason for it possessing the highest ACE inhibitory ability. Given that synthetic ACE inhibitors can cause certain adverse effects, such as coughing, taste disturbances, allergic reactions, and skin rashes [42], natural ACE inhibitors may serve as potential substitutes or adjuncts to synthetic drugs in the future.

### 3.6. Correlation Analysis

**Figure 5:**
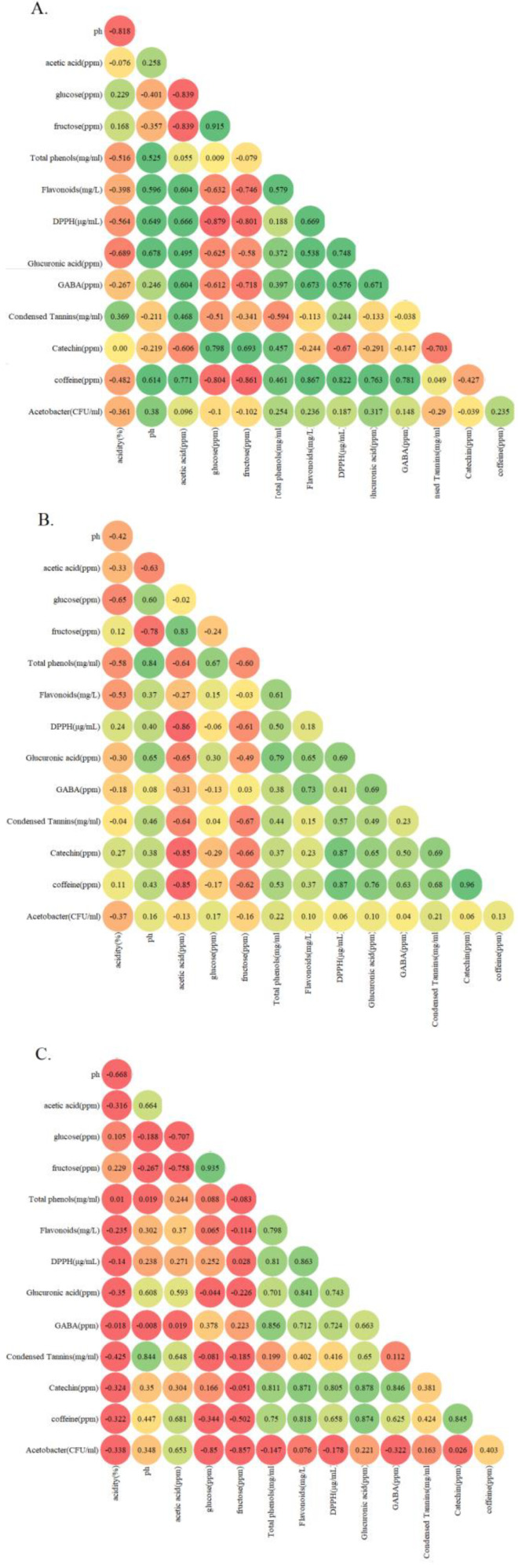
Pearson correlation analysis between the nutritional and bio-functional compounds and antioxidant capacity. (**A**) Lightly oxidized tea beverage. (**B**) Moderately oxidized tea beverage. (**C**) Fully oxidized tea beverage. The indicators in the circle are color-coded to indicate a positive correlation (green), negative correlation (red), or low correlation (yellow) between them. The intensity of the color indicates the strength of the correlation coefficient, with darker shades indicating a stronger correlation.

The pH and catechins of both the LOT and MOT were negatively correlated (r = −0.211, −0.46, respectively) (Figure 5A,B). However, the pH and catechins of the FOT showed a strong positive correlation (r = 0.844) (Figure 5C). This may be related to the fact that the concentration of organic acids in the LOT and MOT increased more than in the FOT during the fermentation process. Figure 5C indicates that the acetic acid bacteria growth in the FOT showed a strong negative association with glucose and fructose (r = −0.85, r = −0.857, respectively). This indicates that the acetic acid bacteria fully utilized the glucose and fructose; the acetic acid and the acetic acid bacteria showed a strong positive association (r = 0.653).

The acetate expression in the LOT was significantly positively correlated with caffeine, GABA, DPPH, and flavonoids (r = 0.771, 0.604, 0.666, 0.604, respectively). The increase in GABA may be related to glucose and fructose. During the process of glycolysis, glucose is converted to pyruvate, and microorganisms can then use glutamic acid dehydrogenase (GDH) and glutamic acid decarboxylase (GAD) to convert pyruvate to GABA [43]. The increase in DPPH activity may be due to the increase in flavonoid contents. The MOT only had a strong positive correlation with fructose (r = 0.83). There was a strong positive correlation between the FOT and Acetobacter, caffeine, condensed tannins, and glucuronic acid (r = 0.653, 0.681, 0.648, 0.593, respectively). The FOT had the strongest correlation (r = 0.811) between catechins and TPC among the three oxidized tea beverages. As catechin is part of the chemical family of flavonoids, which account for approximately 75% to 80% of the polyphenol content of the tea, this indicates that the catechins in FOT have the highest proportion of TPC. Regarding the correlation between TPC and TFC during LOT fermentation, the LOT showed a moderate positive association between TPC and TFC (r = 0.579). The MOT also indicated a moderate positive association (r = 0.609), and the FOT showed a strong positive association (r = 0.798). The correlation between the LOT flavonoids and DPPH was strong (r = 0.669), but there was no correlation between TPC and DPPH (r = 0.188). The correlation between TPC and DPPH in the MOT showed a moderate positive association (r = 0.501), but the correlation between flavonoids in the MOT and DPPH was not associated (r = 0.181) (Figure 5B). This means that the increase in DPPH during the fermentation process may arise from other intermediates with antioxidant properties after the flavonoids are metabolized. The FOT showed a very strong positive association for both the (TFC and DPPH) and (TPC and DPPH) correlations (r = 0.863, r = 0.810, respectively); this means that the DPPH scavenging ability of FOT is mainly derived from TPC and TFC. The functional components and antioxidant capacity in the LOT, MOT, and FOT were positively correlated with each other, indicating an increasing trend during the acetic acid fermentation process.

## 4. Conclusions

In this study, Chin-shin oolong teas with three oxidation levels of lightly oxidized tea, moderately oxidized tea, and fully oxidized tea were added to EGS and fermented for ten weeks. *A. pasteurianus* LYC1783 was isolated and identified through this fermentation process. This study’s findings include that LOT fermentation can obtain the highest catechins among functional and antioxidant components. MOT can obtain the highest glucuronic acid, tannins, total phenols, flavonoids, and ACE inhibitory activity. FOT can obtain the highest GABA. In addition, the free radical scavenging ability of the LOT and MOT increased significantly after fermentation. However, the GABA in the MOT and LOT was much higher than that in the FOT in terms of functional ingredients. In contrast to the current kombucha that mostly uses a black tea decoction and sugar as raw ingredients, this study used EGS that was fermented with lightly or moderately oxidized Chin-shin oolong tea leaves, thereby providing the features of low sugar content, high nutritional and functional values, and high antioxidant and ACEi capacity. It may be considered a novel Kombucha as a potent natural and healthy beverage.

## Data Availability

The datasets generated during the current study are available from the contributor on reasonable request.
